# Regulators of the Proteasome Pathway, Uch37 and Rpn13, Play Distinct Roles in Mouse Development

**DOI:** 10.1371/journal.pone.0013654

**Published:** 2010-10-27

**Authors:** Amin Al-Shami, Kanchan G. Jhaver, Peter Vogel, Carrie Wilkins, Juliane Humphries, John J. Davis, Nianhua Xu, David G. Potter, Brenda Gerhardt, Robert Mullinax, Cynthia R. Shirley, Stephen J. Anderson, Tamas Oravecz

**Affiliations:** Lexicon Pharmaceuticals, Inc., The Woodlands, Texas, United States of America; University of Giessen Lung Center, Germany

## Abstract

Rpn13 is a novel mammalian proteasomal receptor that has recently been identified as an amplification target in ovarian cancer. It can interact with ubiquitin and activate the deubiquitinating enzyme Uch37 at the 26S proteasome. Since neither Rpn13 nor Uch37 is an integral proteasomal subunit, we explored whether either protein is essential for mammalian development and survival. Deletion of Uch37 resulted in prenatal lethality in mice associated with severe defect in embryonic brain development. In contrast, the majority of Rpn13*-*deficient mice survived to adulthood, although they were smaller at birth and fewer in number than wild-type littermates. Absence of Rpn13 produced tissue-specific effects on proteasomal function: increased proteasome activity in adrenal gland and lymphoid organs, and decreased activity in testes and brain. Adult Rpn13^−/−^ mice reached normal body weight but had increased body fat content and were infertile due to defective gametogenesis. Additionally, Rpn13^−/−^ mice showed increased T-cell numbers, resembling growth hormone-mediated effects. Indeed, serum growth hormone and follicular stimulating hormone levels were significantly increased in Rpn13^−/−^ mice, while growth hormone receptor expression was reduced in the testes. In conclusion, this is the first report characterizing the physiological roles of Uch37 and Rpn13 in murine development and implicating a non-ATPase proteasomal protein, Rpn13, in the process of gametogenesis.

## Introduction

The Rpn13 protein, previously termed adhesion-regulating protein 1 or GP110, has been identified as a tumor-associated gene product [1], [2], [3], [4], [5], [6], and more recently as an amplification target in ovarian cancer [7]. Initially, Rpn13 was suggested to regulate cell adhesion as a membrane-associated protein [2], but further examination revealed that it is primarily a cytosolic protein associated with proteasomes [8]. Proteasomes are central molecular complexes of the non-lysosomal, ubiquitin-ATP-dependent protein degradation pathway. Independent studies demonstrated direct interaction of Rpn13 with ubiquitin and the deubiquitinating enzyme Uch37 (also termed UchL5) [9], [10], as well as with the proteasome subunit Rpn2/S1 [11], [12]. Binding of Rpn13 to Uch37 increases the isopeptidase activity of Uch37; therefore it may facilitate the rescue of ubiquitinated substrates from proteolysis [9], [10], [13]. Thus, Rpn13 is emerging as a potentially important docking/coupling protein with a regulatory function in recognition and disassembly of ubiquitinated proteins at the proteasome.

Most of the reports on the physiological significance of Rpn13 and Uch37 function concluded that neither protein was essential for cell survival. Inactivation of all known ubiquitin receptors, including Rpn13, in yeast cells did not induce cell death [12]. Likewise, expression of the *Uch37* homologue, *Uch2*, in fission yeast was not required for survival [14]. Viability of the human HeLa cell line, was also unaffected by knocking down expression of *Rpn13* [15] or *Uch37* [16]. Overexpression of *Rpn13* was reported to increase the adherence of 293T cells [2], as well as human natural killer (NK) and T cells [8], to endothelial cells. However, one study found that overexpression of the C-terminal domain of Rpn13 that modifies Uch37 function, led to cell death in the 293T cell line [10]. To our knowledge, only Rpn13 was studied in a higher organism, the frog, where the *Rpn13* homologue, *Xoom* was indispensable for survival of frog embryos [17].

Biochemical measurements reported to date are not conclusive about the contribution of Rpn13 and Uch37 to the function of the proteasome. In HeLa cells, knockdown of *Rpn13* did not affect the amount of proteasome, degradation of proteins, or the accumulation of ubiquitinated proteins [15]. In sharp contrast, *Rpn13* siRNA decreased proteasome function in 293T cells and increased the ubiquitinated protein content, however overexpression of Rpn13 had a similar effect [10]. *Uch37* knockdown in the same cell line led to decreased deubiquitination activity [9], [10] and expression of the C-terminal domain of Rpn13, that competes for the binding to Uch37, reduced proteolytic activity [9], [10].

We generated *Rpn13* and *Uch37* knockout (KO) mice and performed comprehensive phenotypic analyses to delineate the role of these interacting proteasomal proteins in mammalian physiology. The results indicate differing roles for Rpn13 and Uch37 in mammalian development and furthermore define a requirement for Rpn13 in gametogenesis.

## Results

### Uch37 and Rpn13 are both essential in early mouse development

**Figure 1:**
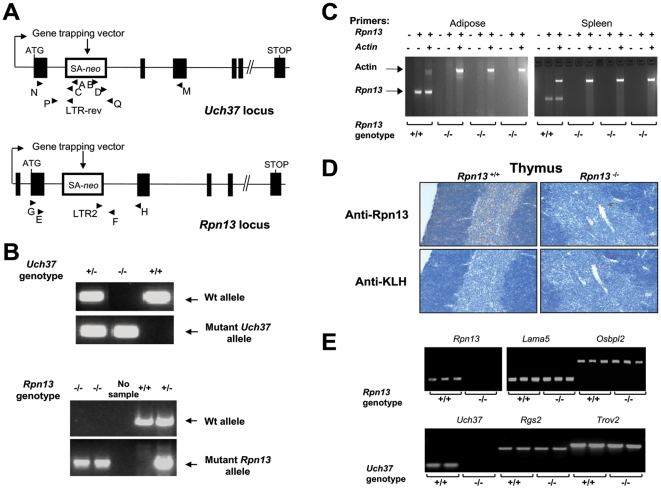
Generation of *Uch37* and *Rpn13* mutant mice. (A) Gene trap mutation and location of primers for genotyping strategy and expression analysis of the *Uch37* and *Rpn13* genes. Arrows and arrowheads indicate transcription start sites. Primers A–D were used to identify the insertion site by nested PCR in the Uch37 clone. Primers E and F flank the genomic insertion site in the *Rpn13* gene and amplify a product for the Wt allele. The LTR2 primer, complementary to the gene-trapping vector, amplifies the mutant allele in conjunction with primer H. SA, splice acceptor sequence; LTR, viral long terminal repeat; Neo, neomycin resistance gene. (B) Genotypic analysis of mice at the *Uch37 and Rpn13* loci was performed by screening genomic DNA isolated from embryos and tail biopsy samples respectively. (C) Indicated gene transcripts were detected in the designated tissues by RT-PCR using primers G and H. (D) IHC was performed using the designated mAbs on the indicated thymus sections. (E) Expression analysis of *Rpn13*, *Uch37* (in 13.5 days old embryos) and their respective neighboring genes. Indicated gene transcripts were detected by RT-PCR.

*Uch37*- and *Rpn13*-null mice were derived from the OmniBank [18], [19] embryonic stem cell clones OST447189 and OST128063, respectively. Inverse genomic PCR of DNA isolated from Uch37 embryonic stem cells revealed that the gene-trap vector had inserted in intron 1 downstream of the first coding exon of the mouse *Uchl5* gene which encodes Uch37 (Figure 1A) and was confirmed by genomic PCR analysis (Figure 1B). The Rpn13 clone carried a gene trap mutation within the second intron of the *Rpn13* gene encoding Rpn13 (Figure 1A). Inactivation of *Rpn13* gene in KO mice was confirmed by genomic PCR (Figure 1B), expression analysis of the gene transcript (Figure 1C), and by immunohistochemical (IHC) staining with a mAb to the Rpn13 protein (Figure 1D). Presence of *Rpn13* transcript was detected only in Wt and not in KO tissues (Figure 1C), while expression of genes immediately flanking *Rpn13* (*Lama5* and *Osbpl2*) was not affected by the *Rpn13* deletion (Figure 1E). The silencing of *Uch37* did not affect the expression levels of *Rgs2* and *Trov2* that flank the *Uch37* gene (Figure 1E).

**Figure 2:**
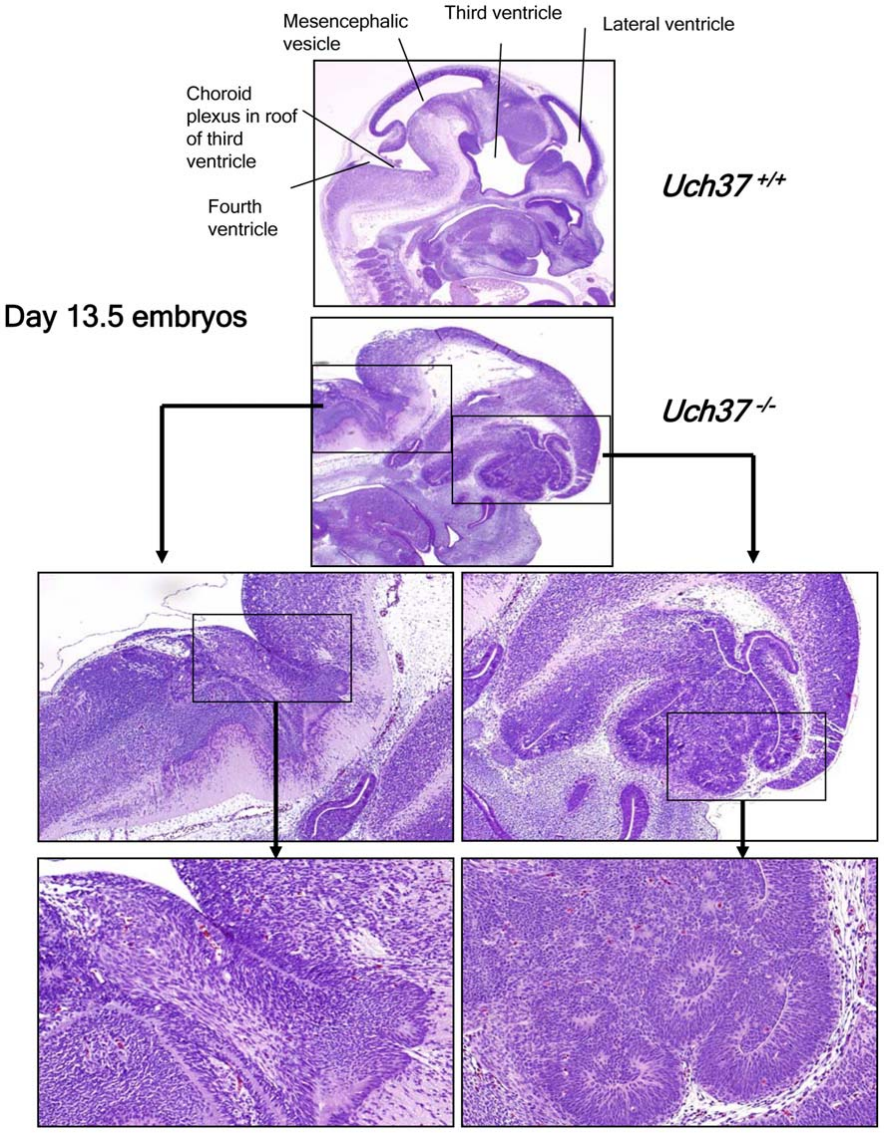
*Uch37*-deficient embryos show defects in brain development. Histological examination of 13.5 days old embryos revealed that, when compared to age-matched Wt embryos, *Uch37^−/−^* mice showed undeveloped lateral and third ventricles in forebrain area, with associated disorganized development of cortex and midbrain as well as undeveloped mesencephalic vesicle and fourth ventricles in midbrain, with disorganized development of cerebellum and hindbrain.

Heterozygous mice (*Uch37* ^+/−^ and *Rpn13* ^+/−^) were fertile and were intercrossed to obtain homozygous progeny. Complete deletion of *Uch37* resulted in prenatal lethality, since no homozygous neonates were identified among 64 pups produced by 10 litters of *Uch37^+/−^* mice. Timed breeding of *Uch37^+/−^* mice showed that *Uch37^−/−^* embryos were underdeveloped and were undergoing resorption from as early as day 8.5. The few *Uch37* ^−/−^ embryos that we were able to analyze at 13.5 days post-conception showed that the decreased viability of mutant mice was most likely due to severe developmental defects of the brain (Figure 2). The developmental lesions in the brain were characterized by the apparent absence of well defined mesencephalic vesicles and lateral ventricles, resulting in a wide range of defects in the formation of the telencephalon, mesencephalon and metencephalon. Disorganized neuronal growth was most evident in the area of the forebrain, cerebellum, and midbrain, with formation of irregular neuronal rosettes and folds in place of the normal periventricular laminae (Figure 2). In contrast, breeding of *Rpn13* ^+/−^ mice resulted in the production of 276 viable homozygous mutant mice out of 1935 progeny, with none displaying any clear developmental neurological lesions. Nevertheless, the ratios of the three possible genotypes at the age of 3 weeks significantly deviated from the normal Mendelian (1:2:1) distribution (29.6% wild-type (Wt; *Rpn13* ^+/+^), 56.2% *Rpn13* ^+/−^, and 14.3% knockout (*Rpn13* ^−/−^); p = 8×10^−27^). This genetic deviation was not due to reduced viability of *Rpn13^−/−^* embryos since examining the embryonic development of *Rpn13^−/−^* mice at 8.5, 10.5, 11.5 and 13.5 embryonic age did not reveal any clear alterations in Mendelian ratios or pathological abnormalities that could explain the reduced number of *Rpn13*-deficient mice (not shown). However, *Rpn13^−/−^* newborns were smaller at birth and as such were less competitive with their Wt and Het siblings for food which could explain their reduced numbers when they reached 3 weeks of age for genotyping. Indeed, we observed that removing the bigger siblings enhanced the chances of *Rpn13^−/−^* mice to survive (data not shown).

### Rpn13 protein exhibits differential expression and function in vivo

**Figure 3:**
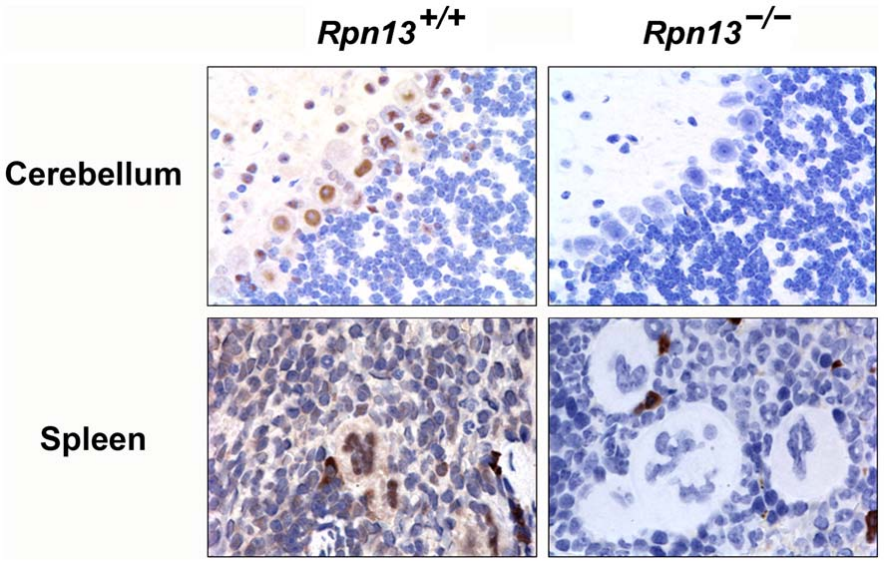
The expression pattern of the Rpn13 protein is cell-type specific. IHC was performed using anti-Rpn13 mAb on the indicated tissue sections.

IHC staining with an anti-Rpn13 monoclonal antibody (mAb) indicated that the Rpn13 protein was present in all Wt tissues examined: thymus (Figure 1D), brain, spleen (Figure 3), adipose, adrenal, cardiac and skeletal muscle, cartilage, gastrointestinal tract, liver, lung, mammary gland, ovary, pancreas, salivary gland, and thyroid (data not shown). However, the staining pattern of Rpn13 was rather cell-type specific as illustrated by the positive staining of Purkinje cells but not the granular layer in the cerebellum and the intense staining in megakaryocytes compared to weak positive staining of the B-cell areas of the spleen (Figure 3). No specific staining was evident in *Rpn13* ^−/−^ tissues (Figure 1D and 3).

**Figure 4:**
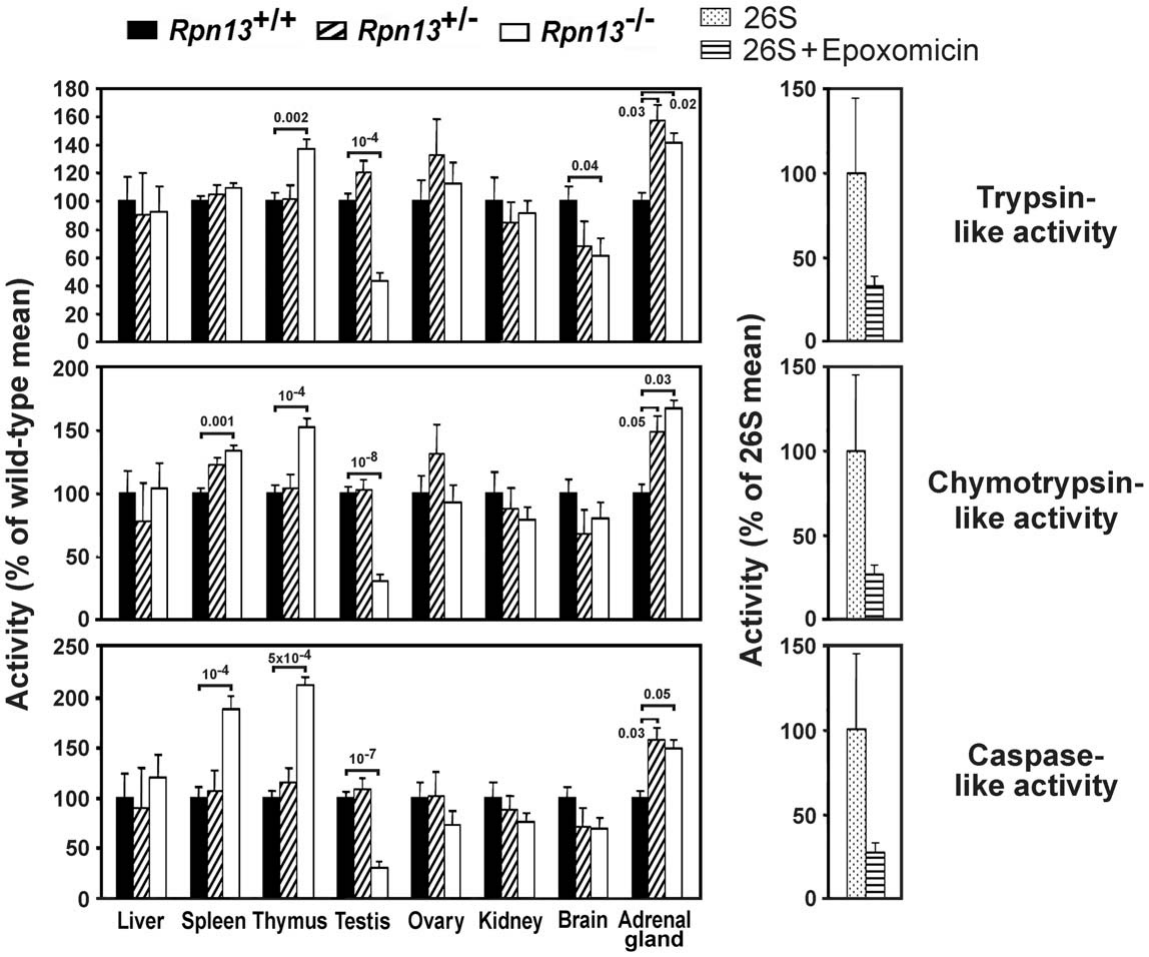
The effect of *Rpn13* deletion on proteasome function is tissue-specific. Indicated proteasome activities were measured in protein lysates prepared from the designated tissues (left panels), or in purified 26S preparations with or without epoxomicin as controls (right panels). For the tissue lysates, data were obtained from 7–19 samples of each genotype pooled from at least four independent experiments giving similar results. 26S controls included 3 samples for each treatment. Enzyme activities were normalized to the mean of the Wt or control values and are presented as mean ± SEM. Numbers above bars indicate P values compared to Wts.

Measurement of Rpn13-associated enzyme activities in KO mice also demonstrated differential, tissue-specific effects of *Rpn13* deletion. Rpn13 has been identified as a component of the proteasome pathway [10], [15]. Therefore we performed comparative measurements of the trypsin-like, chymotrypsin-like, and caspase-like activities of the proteasome in tissue extracts from *Rpn13* ^−/−^, *Rpn13* ^+/−^ and *Rpn13* ^+/+^ mice. All proteasome functions were significantly decreased in the testes of *Rpn13* ^−/−^ mice when compared to Wt littermates (Figure 4). On the other hand, the same activities were significantly increased in the adrenal gland and thymus, and with the exception of trypsin-like activity, in the spleen of *Rpn13* ^−/−^ mice. Brain tissues from *Rpn13* ^−/−^ mice showed a modest decrease in proteasome function but reached statistical significance only for trypsin-like activity (Figure 4 upper panel). In contrast, proteasomal activities in the liver, ovaries, and kidneys were not affected by the *Rpn13* deletion.

### Rpn13 is indispensable for normal spermatogenesis and oogenesis

**Figure 5:**
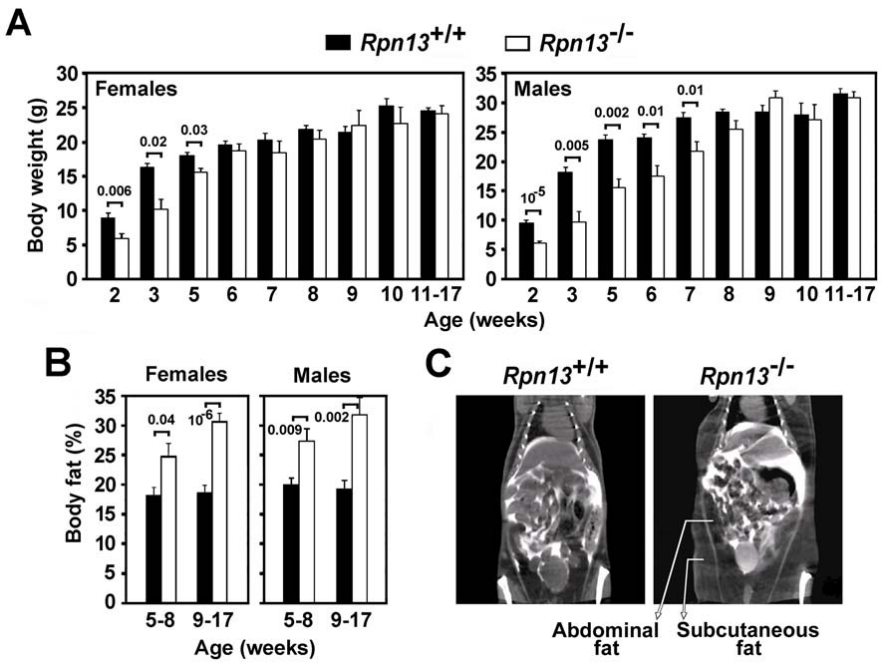
*Rpn13* deficiency affects body weight and composition. Body weights (A) and composition (B) were measured for the indicated mice at the time points and intervals shown. Minimum 3, maximum 38 mice were studied at each time point. Numbers above bars indicate P values compared to Wts. (C) Representative images of CAT-scan analysis of 16-weeks old male mice with the indicated genotype. An additional male and a female *Rpn13* ^−/−^ mouse were also analyzed along with Wt littermates and showed similar increases in fat deposits as marked on the image.

*Rpn13* ^−/−^ mice, along with their Wt littermates, were subjected to our standard phenotypic evaluation protocol, which is an integrated suite of medical diagnostic procedures designed to assess the function of the major organ systems (described in detail in ref. [19]). The *Rpn13* ^−/−^ mice were smaller than their Wt littermates during the first weeks of life, but both sexes reached normal body weight by 6–8 weeks of age (Figure 5A). Independent of body weight, gender, or age, all *Rpn13* ^−/−^ mice had significantly higher body fat content detected by NMR analysis (Figure 5B) and dual energy X-ray absorptiometry (DEXA) (data not shown). CAT-scan also demonstrated increased abdominal and subcutaneous fat depots in *Rpn13* ^−/−^ mice (Figure 5C).

**Figure 6:**
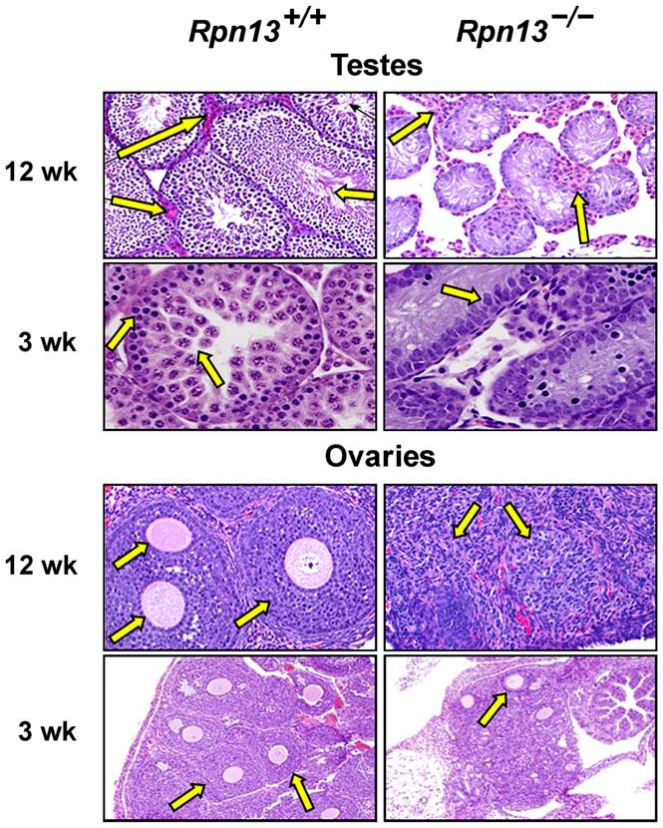
Absence of Rpn13 results in abnormal sexual development. Testes and ovaries from mice of indicated genotype and age were subjected to H&E staining. Arrows point to the following features: 1. Maturing sperms in the tubular lumen of the *Rpn13* ^+/+^ testis which are absent in the *Rpn13* ^−/−^ section, and Leydig cell clusters present in both sections of 12-weeks old mice; 2. Spermatids and spermatogonia in the *Rpn13* ^+/+^ testis are largely missing from *Rpn13* ^−/−^ mice, and hypertrophic Sertoli cells in the *Rpn13* ^−/−^ testes of 3-weeks old mice; 3. Primordial follicles with ova and granulose cells present only in the *Rpn13* ^+/+^ ovary, and replaced by interstitial stroma in the *Rpn13* ^−/−^ ovary of 12-weeks old mice; 4. Ova which are numerous in Wt and few in *Rpn13* ^−/−^ mice at 3-weeks of age.

*Rpn13* ^−/−^ mice are infertile since multiple attempts to breed either male or female mice failed. Histological analysis of 12 weeks old *Rpn13* ^−/−^ male mice revealed hypoplasia of seminiferous tubules and azoospermia (Figure 6). Importantly, the interstitial cells (Leydig cells) (Figure 6, upper panels), accessory sex glands, and secondary sex characteristics in the salivary glands and kidney (data not shown) were all normal in appearance. In 3 week old males, the seminiferous tubules of Wt mice contained numerous pachytene spermatids and spermatogonia, while in *Rpn13* ^−/−^ mice the seminiferous tubules were lined predominately by hypertrophic Sertoli cells, and most of the identifiable spermatids were early stage and undergoing apoptosis (Figure 6).

In 3 week old females, ovaries of Wt mice contained numerous ova surrounded by deep layers of granulose cells, and numerous primordial follicles were present. In contrast, in young female *Rpn13* ^−/−^ mice there were relatively few ova and those present were surrounded by a single layer of cells. Primordial follicles could not be reliably identified in routine hematoxylin and eosin (H&E) stained sections from 3-week old *Rpn13* ^−/−^ mice, but other reproductive tract structures and secondary sex characteristics appeared to be within normal limits (data not shown). In 12-week old *Rpn13* ^−/−^ females, there were no detectable primordial ovarian follicles or corpora lutea, and there were only a few secondary atretic and cystic follicles present within the vacuolated interstitial ovarian stroma when compared to Wt littermates (Figure 6, lower panels).

### Rpn13 deficiency leads to altered T-cell development

**Figure 7:**
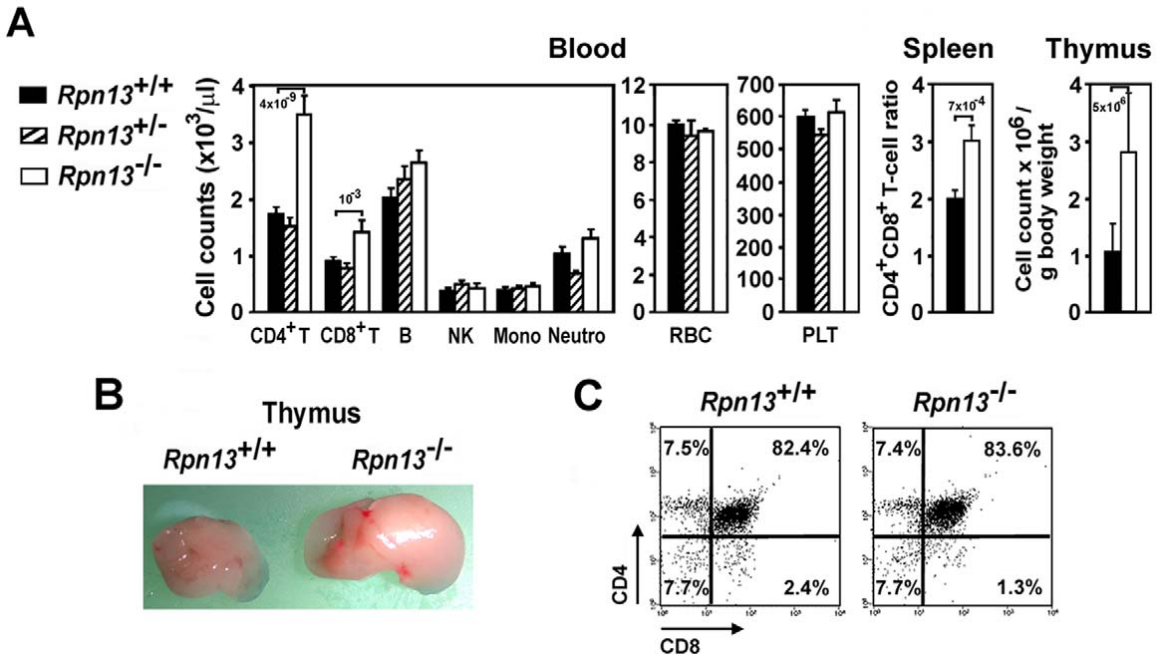
Deletion of *Rpn13* alters T-cell development. (A) Hematopoietic cell profile of the indicated tissues harvested from mice with genotypes designated on the figure. Data were obtained from 4–5 month old mice (n = 15–31) and are presented as mean ± SEM. Numbers above bars indicate P values compared to Wt mice. Mono, monocytes; Neutro, neutrophils. (B) Representative picture of thymi from mice of indicated genotype demonstrating difference in size of the organs. (C) Representative flow cytometric plots show normal CD4/CD8 mAb staining patterns of thymocytes from mice of both genotypes. Samples from 10 additional mice of each genotype gave similar results.

Decreased gonadal function and alterations in steroid or growth hormone levels can impact immune system development and function, particularly thymic output [20], [21], [22]. Since the overall phenotype of *Rpn13* ^−/−^ mice was consistent with disturbed hormonal balance in the KO animals, we performed blood cell and immune tissue analysis. *Rpn13* ^−/−^ mice harbored significantly higher number of CD4^+^ T cells and, to a lesser extent, CD8^+^ T cells in the peripheral blood when compared to Wt littermates (Figure 7A). In contrast, B cells, NK, monocytes, neutrophils, red blood cells and platelets (PLT) were present at comparable levels in the two genotypes. Correspondingly, FACS analysis of splenocytes also showed a significant increase in the CD4/CD8 T-cell ratio (Figure 7A). Consistent with the above findings, *Rpn13* ^−/−^ mice developed larger thymuses with more cellularity compared to Wt mice (Figure 7A and B). However, the increased number of thymocytes displayed normal distribution of cells in the main developmental stages of CD4/CD8 double negative (DN), double positive (DP), and single positive (SP) cell subsets (Figure 7C). The increased thymocyte development was not due to clonal expansion of specific thymocyte subsets since examination of the repertoire of TCR α- and β-chain variable regions showed similar distribution in *Rpn13* ^−/−^ and Wt thymocytes (Figure S1). Similarly we did not observe any differences in the proliferation of thymocytes to anti-CD3 plus IL-2 and Concanavalin A or their susceptibility to apoptosis by anti-CD3 induced deletion in vivo and Dexamethasone in vitro that would explain the increase in thymocyte population or the accumulation of CD4 T cells (not shown). Consistent with the above findings, *Rpn13* ^−/−^ did not develop signs of autoimmune disease or thymomas during the course of our studies (up to 6 month of age).

### Rpn13 is essential for normal hormonal balance

**Figure 8:**
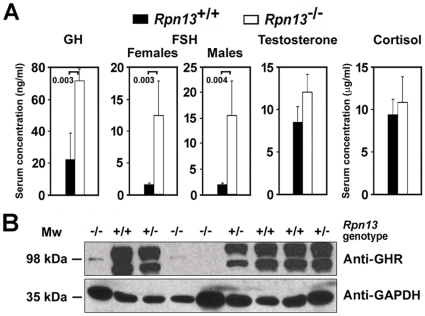
Rpn13 is essential for normal hormonal homeostasis. (A) Serum concentration of the indicated hormones was measured in the mice designated on the figure. Data were pooled from at least three independent experiments giving similar results. Minimum 4, maximum 24 mice were studied in each group, and only male mice were used for testosterone measurements. Data are presented as in Figure 7A. (B) Expression of GH receptor and control GAPDH in the testes of mice with the indicated genotype. Western blot analysis was performed with the antibodies designated on the figure.

We next tested whether the observed phenotype of *Rpn13* ^−/−^ mice is associated with changes in the levels of hormones involved in body growth and composition, sexual maturity, and thymic development. Circulating levels of GH and FSH were significantly elevated in the serum of *Rpn13* ^−/−^ mice compared to Wt animals (Figure 8A). No apparent differences were observed in serum concentrations of testosterone or cortisol between the two genotypes. We further tested expression of GH receptor in protein lysates of testes (Figure 8B), ovaries, thymus, and adrenal glands (data not shown). The testes of *Rpn13* ^−/−^ mice showed severe reduction in GH receptor protein in comparison to Wt levels while the other tissues examined did not demonstrate significant differences in GH receptor expression.

## Discussion

Recent biochemical and genetic experiments identified the Uch37 and Rpn13 proteins as novel proteasomal interacting partners [9], [10]. However, the functional role of these proteins in the regulation of proteasomal activity is unclear. Knockdown studies provided inconsistent results for readouts of either survival or proteasome function in yeast and mammalian cell lines [9], [15], [23]. Therefore we explored the physiological consequences of loss of function of Uch37 and Rpn13 in mice. The KO phenotypes demonstrated that, unlike Rpn13, Uch37 is essential for prenatal development and survival, which implies different functions for these two proteins *in vivo*. This is not unexpected, since both Uch37 and Rpn13 have been implicated in distinct molecular pathways presumably independent of their mutual interaction. For instance, Uch37 can also associate with Rpn10 (S5a) at the proteasome complex [10], [14], as well as with the human Ino80 chromatin-remodeling complex [24] and with Smad proteins, particularly with Smad7 [25]. Of note, *Rpn10* deletion results in early-embryonic lethality in mice [26]. Thus, it is possible that disruption of one or more of these additional pathways leads to lethality of the *Uch37^−/−^* mice. On the other hand, Uch37 may remain active in the proteasomal complex and continue to regulate protein deubiqutination in the absence of Rpn13. *Rpn13^−/−^* mice showed only reduced viability and close to 60% of the surviving pups reached adulthood, which enabled us to further interrogate the *in vivo* function of this proteasomal receptor.

Surviving *Rpn13* ^−/−^ mice showed defective gametogenesis, delayed growth, high fat accumulation, and altered T-cell development. Collectively, these abnormalities are consistent with dysregulation of hypothalamic-pituitary-gonadal axis encompassing key hormonal pathways. Absence of Rpn13 expression may have affected endocrine circuits directly or indirectly at multiple locations since altered proteasomal function was detected in various (but not all) organs, including testes, brain, adrenal gland, and lymphoid tissues.

The phenotype of the *Rpn13* KOs and analysis of proteasome activities in different tissues of *Rpn13* ^−/−^ mice suggest a relatively tissue-specific physiological role for this proteasome receptor. Depending on where it is expressed, Rpn13 could play enhancer, repressor, or non-critical role in regulating proteasome activity. Contrasting activity profiles of proteasomal proteins in different tissues is not uncommon, and is thought to reflect tissue-specific diversity of proteasomes. Indeed, Rpn13 is not a constitutive proteasome subunit, since only a fraction of 26S proteasomes contain Rpn13 [9], and we observed significant cell type-specific differences in the expression pattern of the protein in certain tissues which might explain the seemingly contradictory data from cell lines about Rpn13 function [9], [10]. Tissue-specific functions for proteasome complex regulators have been reported before. PA700/19S, PA28α/β, and PA200, are molecules that associate with the α heptameric rings of the 20S and regulate its functions [27], [28], [29]. Even though these proteasomal proteins are broadly expressed, they play different physiological roles in various tissues: PA200 is required for normal spermatogenesis [30] while PA28α and PA28β modulate immunoproteasome function [31]. The developmental abnormalities in *Rpn13^−/−^* mice were observed in a number of tissues, and it is likely that the absence of Rpn13 not only impacted organ development directly by altering the proteasome function in those tissues, but also had an indirect effect via the neuroendocrine pathway.

*Rpn13* KOs registered elevated serum concentrations of two key hormones, GH and FSH. The testes of *Rpn13^−/−^* mice also showed reduced levels of the GH receptor, whose expression was shown previously to be modulated by proteasome function [32]. The GH-GH receptor interaction is known to have pleiotropic effects on growth, metabolism and sexual maturation [33]. Although dysregulation of GH signaling via GH receptors is a likely contributor to the phenotype of *Rpn13^−/−^* mice, it cannot account for all aspects of it. The defect in sexual maturation of *Rpn13^−/−^* mice is more severe than that of “GH-resistant” mice, which are deficient in GH receptor expression and also register elevated levels of GH [34], [35], [36]. However, the observed changes in T-cell development in *Rpn13^−/−^* mice are highly consistent with increased GH levels and signaling via GH receptors expressed in the thymus. Growth hormone enhances thymopoiesis and augments thymus volume in both rodents and humans [37]. Moreover, it stimulates the selective trafficking of CD4^+^ thymic emigrants compared to CD8^+^ cells to the periphery, leading to increased CD4/CD8 ratio in the lymphoid organs [37]. All these effects parallel the increased thymic cellularity and overrepresentation of CD4^+^ cells that we found in the *Rpn13* ^−/−^ mice. Besides GH-related effects, Rpn13 may also directly influence thymus development, since we observed increased proteasome function in the thymus of *Rpn13* ^−/−^ mice. In T cells, proteasomes regulate activation, proliferation [38], [39], and expression of apoptotic factors [40]. However, transfer of *Rpn13* ^−/−^ bone marrow cells into irradiated Wt mice did not reproduce the KO immunological phenotype (unpublished observations), which strongly suggests that the alteration in thymus size and the accumulation of CD4^+^ T cells in the periphery are not intrinsic to T-lineage cells but likely mediated by external factors, such as GH.

Perturbations in ovarian-pituary interactions and lack of gonadal feedback from certain endocrine tissues may also explain the elevation of FSH levels in the *Rpn13^−/−^* mice. For instance, endocrine peptides, including inhibin, which suppresses pituitary FSH production, are synthesized and released from the ovary and testis in mammals. Absence of inhibin in KO mice leads to high circulating levels of FSH [41].

Recently, Rpn13 was shown to be significantly upregulated in the early stages of ovarian cancer and continues to increase as the disease progresses. This increase has been correlated with highest recurrence and metastasis [7]. Importantly, the most striking phenotype in female *Rpn13^−/−^* mice was a developmental defect in the ovaries. However, deletion of Rpn13 did not affect the overall proteasomal activity in the ovaries themselves, suggesting that the underlying mechanism of defective gametogenesis in the *Rpn13* KO mice may not be intrinsic to this reproductive organ but likely has neuroendocrine origin. Alternatively, since Rpn13 is differentially expressed by different cell types in the same tissue, it is possible that certain cells that regulate organ development were impacted by its absence more than others and this would not be reflected by studies which measure proteasome function at the organ level.

In conclusion, our results demonstrate that although Uch37 and Rpn13 have been shown to associate in many studies [9], [10], [13], their physiological roles may differ considerably. Uch37 plays a vital role in brain development early in the embryonic phase while Rpn13 is required mainly after birth for proper growth and maturation of the reproductive and endocrine systems. The developmental defects and abnormalities in the reproductive and immunological systems in *Rpn13^−/−^* mice likely involve pleiotropic effects and feedback mechanisms intrinsic to the neuroendocrin pathways. Further studies aimed toward identification of proteins whose ubiquitination patterns are affected by deletion of Rpn13 and Uch37 could be informative for identification of factors leading to the observed KO phenotypes and possibly affecting tumor development. A unique characteristic of Rpn13 protein is that its absence completely blocks oogenesis and spermatogenesis apparently without affecting secondary sex characteristics. Investigation of the potential effect of inhibiting Rpn13 in cancer is warranted, since it may provide avenues to adjunct therapy of ovarian tumors or seminomas with less disruption to estrogen or testosterone levels.

### Note Added to Proof

A recent publication has provided further proof of the selective role of Rpn13 in proteasomal function by identifying nitric oxide synthase and IkappaB-alpha as two specific substrates of the Rpn13/Uch37 complex [42].

## Materials and Methods

### Generation of *Uch37* and *Rpn13* mutant mice

OmniBank ES clones [18], [19] were selected for microinjection into host blastocysts based on sequence similarity to the mouse *Uch37* and *Rpn13* genes (accession numbers NM_019562 and NM_019822.3, respectively). The precise genomic insertion sites of the gene-trapping vectors were determined by inverse genomic PCR as described [43]. Mutant mice were generated by using standard methods [44]. For *Uch37*, two rounds of PCR using nested primers complementary to the gene trapping vector (Round 1: A: 5′- GTT AAG ATC AAG GTC TTT TCA CCT GGC -3′ and B: 5′- CCA TAT TCA GCT GTT CCA TCT GTT CC -3′; Round 2: C: 5′- GCC TCG ATC CTC CCT TTA TCC AGC -3′ and D: 5′- AAA TGG CGT TAC TTA AGC TAG CTT GC-3′) were used to amplify vector-genomic junction sites. Genotyping of mice was performed by using quantitative PCR [43]. Wt and mutant *Rpn13* alleles were amplified with oligonucleotide primers E (5′-GAA AGA CAG GAC CTC TGG GAC CGT - 3′), F (5′-CTG GCT AGC TGC TCC TAA GTG TAA-3′), LTR2 (5′-AAA TGG CGT TAC TTA AGC TAG CTT GC-3′, and H (5′-GCC GCT TGG ACC CTG CCT TAA AC-3′). Wt and *Uch37* mutant embryos were genotyped using P (5′-TTA ATG TGA ATA ATC GGA ATG CTG G-3′) and Q (5′-CTA TAC AAT ATG GTA TCT GAT TTG G-3′) to detect the Wt gene. P and LTR-rev (5′-ATA AAC CCT CTT GCA GTT GCA TC-3′) were used to detect the mutant *Uch37* alleles.

### Animal background and care

All mice analyzed were maintained in an AAALAC-accredited animal facility at Lexicon Pharmaceuticals, Inc. (accreditation unit #001025). Mice were housed in a barrier facility at 22°C on a fixed 12-hour light and 12-hour dark cycle with free access to water and standard rodent chow (9F 5021; Purina, St Louis, MO). Procedures involving animals were conducted in conformity with the Institutional Animal Care and Use Committee guidelines that are in compliance with the state and federal laws and the standards outlined in the Guide for the Care and Use of Laboratory Animals. All experiments were approved by the Institutional Animal Care and Use Committee of Lexicon Pharmaceuticals, Inc. (approved protocol permit numbers 054, 125 and 162). Unless otherwise indicated, all experiments were carried out on 10- to 16-week-old mice of mixed genetic background (129/SvEvBrd and C57BL/6J) representing both sexes of littermate mutant and Wt animals. For presentation of data, the age of mice was rounded to the nearest week.

### Expression analysis for gene transcripts

Total RNA was extracted from mouse tissues using a bead homogenizer (BioSpec Products, Inc., Bartlesville, OK) and Trizol reagent (Invitrogen, Carlsbad, CA) according to the manufacturer's instructions. Reverse transcription (RT) was performed to produce cDNA using 5 µg total RNA with SuperScript II (Invitrogen) and random hexamer primers, according to the manufacturer's instructions. The same amount of water was substituted for enzyme as control for genomic contamination. PCR amplification was performed using 1 µl of the RT product at initial denaturing step of 95°C for 2.5 min followed by 30 cycles of 95°C (30 sec), 59°C (20 sec), 70°C (1 min) with oligonucleotide primers complementary to Rpn13 (G: 5′-ACT ACG GTC ACC CCA GAT AAA CGG AAA G-3′and H), *Uch37* (N: 5′- TGT CTC ATG GAA AGC GAC CC-3′ and M: 5′- GCC ACT TGA AAA GAA AAA TTA ACC C-3′), *Rgs2* (5′- GAT TGG AAG ACC CGT TTG AGC TAC T-3′ and 5′- TGG GCA ATC AGA GAT TTC GTT TGG A-3′) and *Trov2* (5′- GAA AGT GAA GCG CAC GAA AGA CGA C-3′ and 5′- GGC ACT GAC GTC AAC AGC GAG CAA G-3′). For analysis of Laminin, alpha 5 (*Lama5*, accession number XM_203796) and oxysterol binding protein-like 2 (*Osbpl2*, accession number NM_144500) expression, 5 µl of a 1:7.5 diluted RT product was used. PCR was performed at an initial denaturation step of 95°C for 2.5 min followed by 35 cycles of 95°C (30 sec), 60°C (30 sec), and 72°C (60 sec) with oligonucleotide primers complementary to *Lama5* (5′- AAT GGG CAG ATC TCT GTT CGT GAA G-3′ and 5′-CAT AGT AGG TGC TGG GCA GTA GGA C-3′) and *Osbpl2* (5′-TGG AGA GAA TGC AGT CTG TCG C-3′ and 5′-CAC TCT GTC CAC TTG CCA TAC ATG A-3′). Primers to the mouse β-actin (GenBank accession number M12481; 5′-GGC TGG CCG GGA CCT GAC GGA CTA CCT CAT-3′ and 5′-GCC TAG AAG CAC TTG CGG TGC ACG ATG GAG-3′ or 5′- GAC CTT CAA CAC CCC AGC CAT GTA-3′ and 5′- GCA CTG TGT TGA CAT AGA GGT CTT TA-3′) and GAPDH (NM_008084; 5′-GGA AAG CTG TGG CGT GAT GG-3′ and 5′-ACC CTG TTG CTG TAG CCG TAT TCA T-3′) genes were also included as internal controls for sample handling. PCR products were run on a 2.5% agarose gel along with a 100 bp ladder. Gene specific bands were verified by nucleotide sequencing.

### Histological analysis

Freshly isolated tissues and embryos were immersion-fixed in 10% neutral buffered formalin, embedded in paraffin, sectioned at 4 µm, and stained with H&E on glass slides (Superfrost Plus; Fisher Scientific, Pittsburgh, PA). IHC on tissues was done as described previously [43]. The anti-Rpn13 mAb used in this study was generated by immunizing *Rpn13^−/−^* mice intraperitoneally with purified GST-Rpn13 protein in Freund's adjuvant. Spleens from high titer mice were harvested following a final intravenous boost with purified GST-Rpn13 and splenocytes were fused with NS1 myeloma cells. Rpn13-specific hybridomas were identified by ELISA screening of hybridoma culture supernatants on immobilized GST-Rpn13 and counterscreened against GST alone to confirm specificity for Rpn13. Rpn13-specific hybridomas were subcloned by limiting dilution to ensure monoclonality. The mAb used in this study was purified from scaled hybridoma culture supernatant by protein A affinity chromatography.

### Proteasome activity assays

Frozen tissues were lysed for 20 min at 4°C in buffer containing 10 mM Tris (pH 7), 25 mM KCl, 1.1 mM MgCl_2_, 0.1 mM EDTA, 35% glycerol, 1 mM DTT, 1 mM sodium azide, 2 mM adenosine triphosphate, 0.5% Triton X-100, and 1× protease inhibitor cocktail (Sigma Aldrich, St. Louis, MO). Lysates were cleared by centrifugation at 13,000 rpm for 10 min at 4°C, and then the protein concentration of supernatants were adjusted to 1 mg/ml based on measurements with a BCA kit (Pierce, Rockford, IL). Proteasome activities were measured using 10 µl aliquots of supernatants in a Proteasome Glo™ assay system (Promega, Madison, WI), according to the manufacturer's instructions. 0.5 µg of purified 26S proteasome (Biomol, Plymouth Meeting, PA) in 10 µl lysis buffer was incubated with and without 50 µM epoxomicin (Sigma Aldrich) for 1 hr at 4°C, and used as negative and positive control, respectively.

### Nuclear magnetic resonance (NMR) and CAT-scan analysis

Non-invasive body fat measurements of anesthetized mice were performed with a Minispec NMR Analyzer (Bruker minispec US, The Woodlands, TX) and a MicroCAT scanner (ImTek, Inc., Knoxville, TN) using the manufacturers' software. For the CAT-scan, mice were injected intraperitoneally with 0.25 ml contrast agent Omnipaque 300 (Nycomed Amersham, Norway).

### Complete blood cell count (CBC) and flow cytometry (FACS)

Blood was withdrawn from the retro-orbital plexus. CBC analysis was performed using a HemaVet 850 FS (Drew Scientific, Inc., Oxford, CT) instrument. For FACS analysis of whole blood, red blood cells were lysed by hypotonic shock, after which mononuclear cells were washed once in FACS buffer (PBS/0.1% BSA/0.1% NaN_3_/2 mM EDTA), stained for 30 min at 4°C in the dark with fluorochrome-conjugated mAbs, and washed prior to analysis. Single-cell suspensions of thymus and spleen were stained as above except that prior to staining, Fc receptors were blocked for 15 min at 4°C with anti-CD16/CD32 mAbs (Fc Block, BD Biosciences, San Diego, CA). Samples were analyzed using a FACSCalibur flow cytometer and CellQuest Pro software (Becton Dickinson Immunocytometry Systems, San Jose, CA).

The following rat mAbs were used in this study (all purchased from BD Bioscience): anti-CD4-FITC, CD8α-PE, CD19-FITC, CD45-PERCP, CD49b (pan-NK)-PE, and anti-TCRβ-APC. In the peripheral blood, B cells were identified as CD19^+^/CD45^+^/TCRβ^−^/CD49b^−^ mononuclear cells (MNC), T cells as CD4^+^ or CD8^+^ and β^+^/CD45^+^/CD19^−^ MNC, NK cells as CD49b^+^/CD45^+^/CD19^−^/TCRβ^−^ MNC, and monocytes as CD11b^+^/CD45^+^/TCRβ^−^/CD19^−^ MNC. The absolute number of each cell subset was calculated by multiplying its fractional representation determined by FACS by the absolute number of MNC identified by CBC.

### Measurement of hormone concentration

Serum was isolated from retro-orbital blood by centrifugation at 2000 rpm for 5 min. Growth hormone (GH), testosterone and cortisol concentrations were measured by ELISA (Alpha- Diagnostics, San Antonio, TX). Serum follicular stimulating hormone (FSH) measurement was performed by Ani lytics, Inc. (Gaithersburg, MD).

### Western blotting

15 µg of protein lysates were electrophoresed on 4–12% Tris-Glycine gradient mini gels and proteins were transferred to nitrocellulose membranes (all from Invitrogen). TBS-Superblock**®** was used to saturate the membranes and to dilute antibodies (Pierce, Rockford, IL). Specific protein bands were identified using rabbit primary antibodies to the GH receptor (Santa Cruz Biotechnology, Santa Cruz, CA) and GAPDH (Bethyl Laboratories, Montgomery, TX), and a secondary goat anti-rabbit antibody conjugated with horseradish peroxidase (Bethyl Laboratories). Membranes were washed in TBS-Tween (25 mM Tris-HCl, pH 7.8, 190 mM NaCl, 0.1% Tween 20) between the incubation periods. Western blots were developed by using an ECL detection system (GE Life Sciences, Pittsburgh, PA) following the manufacturer's directions.

### Assessment of T cell receptor (TCR) repertoire

TCR diversity in thymocytes and splenocytes was assessed as previously described [45].

### Statistical analyses

Statistical analysis of the offspring ratios of genotypes employed chi-square test. For other data, statistical significance of group differences was evaluated by the unpaired, two-tailed, Student's t test. A p value of <0.05 was considered significant.

## Supporting Information

Figure S1 Analysis of the TCR alpha and beta chain repertoire does not indicate clonal expansion of specific thymocyte subsets in Rpn13^−/−^ mice. RNA extracted from the indicated tissues of mice with the designated genotype was subjected to RT-PCR specific to the TCR-alpha and TCR-beta variable regions shown on the figure.
(0.42 MB TIF)
