# Supplementary figures and images for: Regulators of the Proteasome Pathway, Uch37 and Rpn13, Play Distinct Roles in Mouse Development
Source: PLoS One. 2010 Oct 27;5(10):e13654. doi: 10.1371/journal.pone.0013654 (PMC2965108; doi:10.1371/journal.pone.0013654)

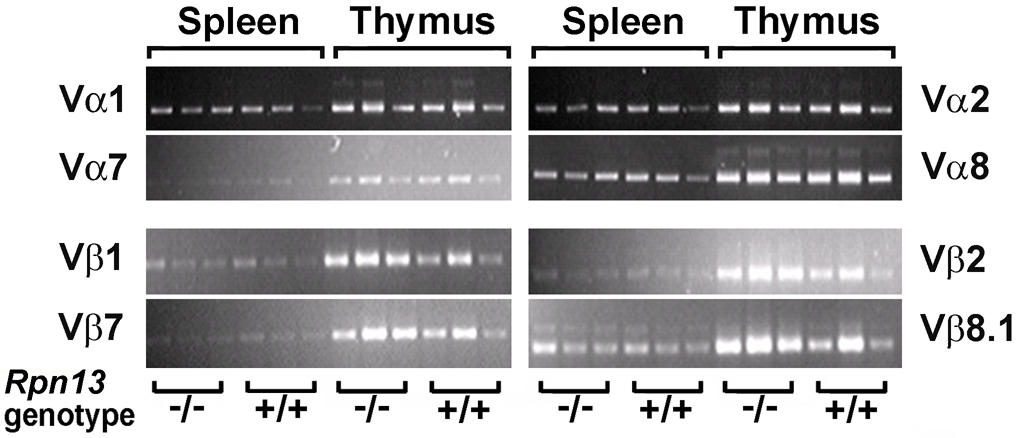

Supplement: Figure S1 — Analysis of the TCR alpha and beta chain repertoire does not indicate clonal expansion of specific thymocyte subsets in Rpn13−/− mice. RNA extracted from the indicated tissues of mice with the designated genotype was subjected to RT-PCR specific to the TCR-alpha and TCR-beta variable regions shown on the figure. (0.42 MB TIF) [file pone.0013654.s001.tif]
